# Clozapine Rechallenge in Treatment-Resistant Schizophrenia: Clinical and Ethical Considerations After Ileus

**DOI:** 10.1192/bjo.2025.10725

**Published:** 2025-06-20

**Authors:** Lily Farakish, Laura Raducu, Anthony Soares

**Affiliations:** 1 Mersey Care NHS Foundation Trust, Liverpool, United Kingdom; 2 Essex Partnership University NHS Foundation Trust, Basildon, United Kingdom; 3 Re:Cognition Health, London, United Kingdom

## Abstract

**Aims:** Clozapine is the cornerstone of treatment for treatment-resistant schizophrenia. It primarily acts by inhibiting dopamine D2 receptors based on the hyperdopaminergic theory of psychosis. Additionally, second-generation antipsychotics (SGAs) interact with serotonin receptors (5-HT2A and 5-HT1A), mitigating extrapyramidal side effects. However, widespread activity on D2 receptors and additional anticholinergic effects can impact gastrointestinal motility, leading to complications such as paralytic ileus. Clozapine has potent anticholinergic activity and is associated with higher risks of paralytic ileus compared with other SGAs.

**Methods:** A male in his late 40s with treatment-resistant schizophrenia, anxiety and panic attacks underwent an elective inguino-sacrotal hernia repair with mesh reconstruction. Psychiatric history was significant for past suicide attempts, including a self-defenestration leading to traumatic brain injury, aggression towards his elderly father and past clozapine-induced neutropenia. He was an ex-smoker. Medications included clozapine 100 mg twice daily, amisulpride 200 mg twice daily, lithium carbonate 625 mg once daily, and hyoscine hydrobromide 300 mcg twice daily.

Postoperatively, the patient developed constipation and abdominal distension consistent with a paralytic ileus. He was placed nil by mouth and managed with nasogastric decompression. During a three-day lapse in antipsychotic treatment on the surgical ward, his mental health deteriorated, presenting with acute psychotic symptoms. The patient lacked insight into his mental health at this time.

Given the failure of alternative antipsychotics previously, the multidisciplinary team (MDT) faced a complex risk-benefit analysis. The potential dangers of reintroducing clozapine, including worsening ileus, were weighed against its irreplaceable role in managing his psychosis, suicidality, and aggression. Ultimately, clozapine was restarted cautiously with haematological and gastrointestinal response closely monitored. Psychosis subsequently improved with no recurrence of ileus, allowing him to continue clozapine treatment.

**Results:** This case highlights the complexities of managing antipsychotic treatment in patients with comorbid physical conditions. Clozapine’s advantage of reducing suicidality and violence were balanced with its potent anticholinergic activity, warranting caution in patients at risk of gastrointestinal complications. The decision to restart clozapine was made after evaluating the significant risks of psychotic relapse. Close MDT monitoring facilitated safe reintroduction, demonstrating necessary case-by-case risk assessments when managing antipsychotics in medically vulnerable patients.

**Conclusion:** Rechallenging clozapine posed significant clinical and ethical challenges, requiring an evidence-based MDT approach. This case underscores the importance of balancing psychiatric needs with medical risks, particularly in treatment-resistant schizophrenia. It also highlights the role of ongoing monitoring and individualised treatment plans in managing complex psychopharmacological decisions. Further studies are warranted to explore safety of clozapine in patients with gastrointestinal-motility disorders.

